# Probabilistic Modeling of Dietary Arsenic Exposure

**DOI:** 10.1289/ehp.1002328

**Published:** 2010-08

**Authors:** Catherine Petito Boyce, Ari S. Lewis, Sonja N. Sax, Barbara D. Beck, Michal Eldan, Samuel M. Cohen

**Affiliations:** Gradient, Seattle, Washington; Gradient, Cambridge, Massachusetts, E-mail: bbeck@gradientcorp.com; Organic Arsenical Products Task Force, Washington, DC; University of Nebraska Medical Center Omaha, Nebraska

We read with interest the article “Probabilistic Modeling of Dietary Arsenic Exposure and Dose and Evaluation with 2003–2004 NHANES Data,” by Xue et al. (2010). We are concerned that the article misrepresented our earlier article on a similar topic (Petito Boyce et al. 2008) and that, by doing so, Xue et al. failed to appreciate the consistency of their estimates of arsenic intake from food and water with ours. Specifically, Xue et al. (2010) stated, “A recent publication [i.e., Petito Boyce et al. (2008)] concluded that typical and high-end background exposures to iAs [inorganic arsenic] in the U.S. population do not present elevated risks of carcinogenicity.” However, they then seemed to call into question our conclusion and to suggest that our analysis either underestimated or failed to include consideration of dietary intake of iAs, citing work by others indicating that iAs intake from food has been estimated to be on the order of several micrograms per day. This suggestion does not accurately reflect our analysis. In fact, our estimates of background exposures to iAs include dietary intake estimates similar to those noted by Xue et al. (2010), and both studies used some of the same data sources.

In our study (Petito Boyce et al. 2008), we conducted a probabilistic analysis using Monte Carlo analysis with Crystal Ball software, incorporating 10,000 iterations, whereas Xue et al. (2010) used the SHEDS model. Table 1 demonstrates the remarkable similarity between the iAs intake estimates from dietary and drinking water sources reported by Xue et al. (2010) and our 2008 intake estimates (Petito Boyce et al. 2008). Our analysis also included estimates of iAs intake from soil, as well as total iAs intake.

A key element of our conclusion (Petito Boyce et al. 2008) regarding the lack of carcinogenic risk was the use of a margin-of-exposure model for iAs, which was applied using an epidemiologically derived no observable adverse effect level. We chose this model based on an analysis of arsenic’s mode of action, from which we concluded that all plausible modes of action were supportive of a nonlinear dose response. Our conclusion was not based on a lower iAs intake estimate, as implied by Xue et al. (2010).

We believe that the analysis by Xue et al. (2010) is important and provides additional understanding of the significance of background exposures to iAs, particularly via ingestion of food. However, by not providing an accurate representation of our work, the authors missed an opportunity to provide additional support for their overall conclusions.

## Figures and Tables

**Table 1:** Comparison of iAs intake estimates.

	iAs intake from food (μg/kg/day)			iAs intake from drinking water (μg/kg/day)		
Study	Mean	50th percentile	95th percentile	Mean	50th percentile	95th percentile
Petito Boyce et al. 2008	0.061	0.048	0.14	0.034	0.001	0.12
Xue et al. 2010	0.05	0.02	0.19	0.025	0.002	0.11
